# Gene Transcription as a Limiting Factor in Protein Production and Cell Growth

**DOI:** 10.1534/g3.120.401303

**Published:** 2020-07-21

**Authors:** Eyal Metzl-Raz, Moshe Kafri, Gilad Yaakov, Naama Barkai

**Affiliations:** Weizmann Institute of Science, Molecular Genetics, Rehovot, Israel

**Keywords:** Transcription, Translation, Protein Burden, RNA polymerase, Cell size, Growth Rate, Yeast, Mediator

## Abstract

Cell growth is driven by the synthesis of proteins, genes, and other cellular components. Defining processes that limit biosynthesis rates is fundamental for understanding the determinants of cell physiology. Here, we analyze the consequences of engineering cells to express extremely high levels of mCherry proteins, as a tool to define limiting processes that fail to adapt upon increasing biosynthetic demands. Protein-burdened cells were transcriptionally and phenotypically similar to mutants of the Mediator, a transcription coactivator complex. However, our binding data suggest that the Mediator was not depleted from endogenous promoters. Burdened cells showed an overall increase in the abundance of the majority of endogenous transcripts, except for highly expressed genes. Our results, supported by mathematical modeling, suggest that wild-type cells transcribe highly expressed genes at the maximal possible rate, as defined by the transcription machinery’s physical properties. We discuss the possible cellular benefit of maximal transcription rates to allow a coordinated optimization of cell size and cell growth.

Cell physiology is characterized by global parameters such as cell growth rate and cell size. These global parameters depend on the integrated function of biochemical, molecular processes that function inside cells to synthesize its various components. Cells coordinate synthesis rates by controlling the fraction of proteins allocated to each process. This allocation is defined by the proteome composition that best optimizes cellular fitness. In microorganisms, maximal growth rates are particularly important for fitness, which is optimized in combination with additional demands such as a rapid response to changing environments. Given these constraints, a fundamental, yet poorly understood question is what restricts the typical values of cell growth rate and size: why do rapidly growing bacteria or yeast divide at time scales of tens of minutes, rather than seconds or days? What sets the typical size of these microorganisms to tens of microns, rather than millimeters or centimeters?

One possibility is that the typical quantitative parameters characterizing cell physiology are set by mechanistic constraints that limit the biosynthesis rates within the cell. Ribosome elongation rate, for example, defines the absolute minimal division time: during balanced growth, doubling time must be long enough to allow one translation complex to reproduce itself. This theoretical upper boundary cannot be realized in cells since the translation of additional endogenous proteins other than ribosomes is required for performing the various cellular activities. Still, the measured growth rate in rapidly growing yeast and bacteria do fall in the range (30–50% deviation) predicted by this theoretical maximum of ribosome-only production (Scott *et al.* 2010; Metzl-Raz *et al.* 2017).

Thus, the mechanistic values of ribosome elongation rate and ribosome size play a fundamental role in setting the typical value, or scale, of cell growth rate. Translation elongation, however, is only one of the biochemical processes that lead to protein synthesis, raising the possibility that additional mechanistic parameters similarly restrict cell physiological parameters.

We hypothesized that, given the fundamental contribution of cell growth rate to cell fitness, processes that contribute to maximizing growth would be driven by evolution to function at their maximal possible capacity. Accordingly, processes that operate at their maximal possible capacity, defined by physical constraints, will fail to increase further when demands increase. In the context of protein expression, limiting processes that function at maximal capacity would fail to fully adapt if, for instance, production demands were to increase. Following this reasoning, we employed cells engineered to express increasing amounts of mCherry proteins as a tool to examine processes that are limiting for protein production in wild-type cells. We rationalized that a failure of limiting processes to adapt in these protein-burdened cells fully would be recognizable based on the cellular regulatory and phenotypic response. For example, if nutrients were limiting, forcing excess protein production would further exacerbate nutrient limitation, and lead to the induction of the known cellular response to this limitation. Similarly, if translation factors were depleted in the mCherry producing cells, it would lead to a response similar to that found in cells deleted of the corresponding translation factors.

We previously showed that cells burdened with excessive protein production grow at reduced rates. By separately burdening the translation or transcription processes, we showed that both perturbations reduce growth, to the extent that depends on the environmental conditions (Kafri *et al.* 2016). During balanced growth, the specific growth rate is set by the proteome fraction dedicated to producing translating ribosomes (Waldron *et al.* 1977; Maaløe 1979; Metzl-Raz *et al.* 2017). We, therefore, examined the burdened-cells’ proteome to distinguish the basis of their reduced growth rate. This analysis revealed four processes that contribute to the growth-rate reduction of burdened cells. First, the mere production of mCherry proteins increases the number of cellular proteins and, accordingly, decrease the proteome fraction dedicated to translating ribosomes. This is mostly a passive effect, in the sense that it happens in the absence of any cellular regulatory change. Second, we observed that burdened cells increase in size in proportion to the burden, and thereby, also increase the levels of their endogenous proteins. This effect is compensatory since it reduces the relative abundance of the mCherry proteins and its passive impact on growth rate (Kafri *et al.* 2016; Jonas *et al.* 2018). Third, mCherry expressing cells increase the fraction of translating ribosomes (reduce their ribosome ’reserves’), as compared to wild-type cells, and by this, more efficiently use their available ribosomal capacity (Metzl-Raz *et al.* 2017). Again, this effect is compensating, moderating the consequences of the increase in mCherry levels. Finally, in some conditions, changes in the overall proteome allocation also contribute to the change in the proteome fraction of translating ribosomes.

In this study, we wished to more directly define the molecular biosynthesis processes that are limiting for protein production, hypothesizing, as described above, that these processes will fail to adapt to the excessive demand for protein production in burdened cells under balanced growth. We analyzed the transcription signature of these cells, as a sensitive probe to internal processes responding to the burden perturbation. This analysis revealed that forcing high mCherry expression altered the gene expression pattern, namely, the relative abundances of different genes. The altered expression pattern most closely resembles that of deletion mutants that lack elements of the general transcription machinery, including the deletion of subunits of the Mediator complex (specifically the Head and Tail sub-complexes), SAGA complex, and the SWI/SNF complex. By contrast, the transcription signature of burdened cells had no apparent resemblance to that of cells deleted of translation factors, including ribosomal components. We confirmed the phenotypic relevance of the correlation between burdened cells and mediator mutants by demonstrating epistatic interactions between the burden and mediator mutants. Of note, only ∼5% of the DNA-bound Mediator localized to the engineered mCherry locus. The relative mediator binding to the rest of the genome was mostly unchanged, refuting the possibility that the burden phenotype resulted from competition for limiting Mediator. Examining the overall absolute transcript abundance, we find that burdened cells increase the amounts of endogenous transcripts, perhaps as a consequence of their larger size. The increase in endogenous transcript abundances was mostly uniform between genes, but the proportional increase failed at highly expressed genes, and genes associated with bursty transcription. This lower relative abundance of rapidly transcribed genes explained the similar transcriptional signature between burdened cells and mediator mutants.

Our results, together with data from the literature and mathematical modeling, suggest that transcription is limiting in wild-type cells growing in standard conditions. We demonstrate that wild-type cells transcribe some genes at rates that are close to the maximal possible rate, as defined by the elongation velocity of the RNA polymerase and its footprint on the DNA. Consequently, transcription rates at rapidly transcribed genes cannot increase further, together with the general transcriptional increase we observed in the burdened cells. To rationalize this finding, we asked what the benefit of maximizing mRNA production could be. Modeling these effects, we suggest that transcribing close to the biochemical limit allows cells to maximize cell size while maintaining the evolutionarily-optimized proteome composition that defines the allocation of proteins between the different cellular functions.

## Material and Methods

### Media and Strains

All strains of *S. cerevisiae* used in this study were constructed on the genetic backgrounds of: BY4741 (MATa his3-∆1 leu2-∆0 met15-∆0 ura3-∆0), BY4742 (MATα; his3-∆1 leu2-∆0 met15-∆0 ura3-∆0), or Y8205 (MATα; his3∆1; LEU2∆0; ura3∆0; can1∆::STE2pr-SP_his5; lyp1∆::STE3pr-LEU2)(Brachmann *et al.* 1998; Tong and Boone 2007) using standard genetic manipulations (see Table S1). Strains were grown in SC medium (Sherman and Miner 2002) or SC medium depleted of a specific nutrient. SC limiting media were prepared from YNB without the relevant nutrient (Low Phosphate medium - ForMedium, CYN0804, Low Nitrogen medium - BD 3101130). Phosphate depleted medium was made by adding phosphate in the form of KH_2_PO_4_ to a final concentration of 0.2mM. The level of potassium was preserved by adding KCl (instead of KH_2_PO_4_) in corresponding amounts. Nitrogen limiting medium was prepared from YNB without amino acids and ammonium sulfate (BD 3101130) by supplementing 50µM of ammonium sulfate and the essential amino acids. The various media’s pH values were: SC = 5.0 (except for low N, where the natural pH was about 4.9).

Deletion and double deletion strains created for validation experiments were derived from BY4741 using the LiAc/SS DNA/PEG method described (Gietz and Woods 2002). In each strain, the gene deleted was replaced with the kanMX cassette (*geneΔ*::*KANMX*) using UPTAG and DNTAG primers as described in the Yeast Deletion Project (http://www-sequence.stanford.edu/group/yeast_deletion_project/usites.html). The deletion was then validated with primers A, B, and kanB.

### Plasmids

p34_TDH3 and p69_TDH3 were crated as described in (Kafri *et al.* 2016). Plasmids and their sequences are available upon request.

### Protein burden libraries creation

Protein burden libraries were generated as described in (Kafri *et al.* 2016). Briefly, the pTDH3-driven mCherry plasmid was integrated into the yeast genome after linearization by the restriction enzyme MfeI. Following selection, single colonies were handpicked to create several hundred candidates. The candidates’ fluorescence levels were measured by flow cytometry. A representative library of the different fluorescence levels (indicating different copies of burden plasmid integration) was then created (each library typically contains tens of strains). Nine copies of the Myc epitope were integrated into the C terminus of Med15,16 and 22 for the generation of the strains used for the ChIP analyses (plasmid pYM21 (Janke *et al.* 2004)).

### Flow cytometry

Flow cytometer measurements and analysis were done using the BD LSRII system (BD Biosciences). mCherry flow cytometry was conducted with excitation at 488nm and emission at 525 ± 25nm for GFP samples. For mCherry markers, excitation was conducted at 594nm and emission at 610 ± 10nm. The average number of cells analyzed was 30,000.

### Competition assays

Cells were grown overnight to stationary phase. A wild-type reference GFP positive strain was then co-incubated with each of the mCherry burden strains at 30°^C^. The initial OD was set to ∼0.05, and the WT initial frequency was ∼50% of the total population. Following growth in the specific condition, the number of generations was calculated from the dilution factor. Frequencies of GFP *vs.* mCherry cells were measured by flow cytometry. The cells were diluted once a day and may have reached a stationary phase. A linear fit of the log_2_ for the WT frequency dynamics was used to calculate the slope for each competition assay. The relative fitness advantage is derived from the slope divided by log_2_. The ‘% of WT division rate (*µ*)’ is 1 + *fitness advantage*. Each strain percentage of *µ-WT* was presented against its mCherry levels from the second day of the experiment or against its copy number calculated from the mCherry levels. Experiments were performed in 96 well plates.

### Epistatic interactions

Epistatic interactions were performed as described previously (Segrè *et al.* 2005). Briefly, we calculated the scaled epistasis between the deletion mutants relative growth rate (Figure S4B) and the burden effect per one integrated copy (1 - the slope of the linear fits (*S*), Figure S4C) according to the equation:

$$\tilde{\varepsilon }=\frac{w_{xy}-w_{x}w_{y}}{\left|{\tilde{w}}_{xy}-w_{x}w_{y}\right|}$$

Where Wx, Wy, Wxy are the burden relative growth rate, deletion mutant relative growth rate, and burden relative growth rate on the background of the deletion mutant, respectively.

Due to the burden’s small effects, we calculated ${\tilde{w}}_{xy}$ as $\min\;\left(w_{x},w_{y}\right)forw_{xy}>w_{x}w_{y}$.

$\tilde{\varepsilon }$ denote the epistatic interaction: $\tilde{\varepsilon }\approx 0$, when there is no epistasis, $\tilde{\varepsilon }\approx -1$ for negative epistasis and $\tilde{\varepsilon }\approx -1$ for positive epistasis.

### RNAseq transcription protocol and analysis

As described in Voichek *et al.*,(2018). Briefly: Cells were grown to OD_600_ of 0.2-0.4 after >6hr in exponential growth and flash-frozen in liquid nitrogen after centrifugation and media removal. RNA was extracted using the Nucleospin 96 RNA kit with modifications for working with yeast. Lysis was performed by mixing the cells with 300 µl lysis buffer [1M sorbitol (Sigma S1876), 100 mM EDTA 0.5 M, and 100 U/ml lyticase]. The lysis mixture was transferred to a 96-well plate that was incubated at 30° for 30 min. The plate was then centrifuged for 10 min at 3000 rpm, and the supernatant was transferred to a 96-well plate provided by the Nucleospin 96 RNA kit, followed by extraction as described in the kit protocol. Labeled cDNA was created from RNA extracts, and cDNA was barcoded and then sequenced in the Illumina HiSequation 2500 system, using a Truseq SR Cluster Kit v3 -cBot-HS cluster kit and a Truseq SBS Kit v3-HS run kit (50 cycles).

### Processing and analysis of sequenced RNA

Processing and analysis of sequenced RNA were as described in Voichek *et al.*,(2018). The analysis was based on the median of 6-8 exponentially growing biological repeats for each genomic copy number (SC/Low N – 8; Low Pi – 6).

### Flocculation assay

Flocculation assays were performed on the background of *med12*Δ as follows: several double deletion strains were created as described above in *Strains* in addition to burden library generated as described above in *Protein burden libraries creation*. Strains were grown overnight at 30° with shaking until saturation. Next, at time point 0, the tubes were strongly vortexed for 30sec following OD_600_ measurement every few seconds, as indicated in Figure 4B. OD values were normalized to time point 0.

### ChIPseq

Cells were grown overnight at 30^oC^, with shaking to ≈OD0.6-0.8. Next, cells were washed in ice-cold PBS without Ca^++^ and Mg^++^ followed by resuspension in 2mM DSG (TS-20593, Rhenium, 50mg DSG in DMSO, PBS without Ca^++^ and Mg^++^) and agitation for 30min at room temperature. 1% formaldehyde was added, and cells were crosslinked for an additional 5 min. The crosslinking was stopped by adding glycine to a final concentration of 125mM and incubating at room temperature for 5 min. Cells were washed twice with ice-cold DDW (3800 rpm, 4^oC^, 2-5 min) and flash frozen. ChIP was performed as in Voichek *et al.*,(2018) using Dynabeads Protein G (Invitrogen) that were incubated overnight with the Myc 9E10 antibody. Cells were resuspended in lysis buffer (50mM HEPESKOH pH = 7.5, 140mM NaCl, 1mM EDTA, 1% Triton X-100, 0.1% sodium deoxycholate with freshly added Protease Inhibitor Cocktail IV (Calbiochem)) on ice and lysed mechanically with zirconium oxide beads in a BBX24-Bullet Blender (Next Advance). Lysates were then sonicated using a Diagenode Bioruptor Plus (35 cycles, high intensity, 30’’ on, 30’’ off). 30µl out of a total of 600µl was taken for Input samples from each lysate. The sonicates were pre-cleared by incubation with Dynabeads Protein G incubated in binding/blocking buffer (PBSx1, 0.5% Tween, 0.5% BSA) for 1 hr at 4^oC^ and subsequently incubated with antibody-coupled beads overnight. Later, lysates were washed on magnet with five rounds on lysis buffer, twice with cold buffer W1 (50 mM HEPES-KOH pH = 7.5, 500 mM NaCl, 1 mM EDTA, 1% Triton X-100, 0.1% sodium deoxycholate), twice with cold buffer W2 (10 mM Tris-HCl pH = 8.0, 250 mM LiCl, 0.5% NP-40, 0.5% sodium deoxycholate, 1 mM EDTA) and twice with cold TE (10 mM Tris-HCl pH = 8.0, 1 mM EDTA). Then lysates were eluted with direct elution buffer (10 mM Tris-HCl pH = 8.0, 1 mM EDTA, 1% SDS, 150 mM NaCl, 5 mM DTT) at 65^oc^, O.N with maximal shaking. Finally, DNA was purified by the addition of 2µl RNaseA (10mg/ml), 37^oc^, 1hr, followed by the addition of 1µl glycogen and 2.5µl Proteinase K (20mg/ml) to each sample, 37^oc^, 2 hr. Proteinase K Inactivation by incubation at 80°^c^ for 20min.

### ChIP libraries

DNA from the previous step subjected to SPRI cleanup with SPRI beads 2.3x and eluted with 10mM Tris-HCL pH 8. DNA libraries for Illumina NextSeq 2500 sequencing were prepared as in Yaakov *et al.* (2017).

### Processing and analysis of ChIP-seq

Reads were aligned to a joined genome of *S. cerevisiae* (SGD, R64-1-1) and pBS69 plasmid. Genomic tracks were created from the sequence reads, representing the enrichment on each position of the joined genome. Physical fragment length was estimated by the shift best aligning the mapped sequenced reads from both ± strands, and single-end sequence reads were then lengthened accordingly (in the range of ≈100-130bp). The signal regions are defined as -500bp before the TSS to the TTS (TSS to TTS coordinates taken from (Xu *et al.* 2009)). Accordingly, the background regions are defined as everything except the signal. Background removal was performed as follows: (1) Each sample was normalized to 1,000,000 reads. (2) Signal and Background regions were defined as above. (3) For each sample, the mean Background value was calculated and subtracted from the entire sample data (Signal and Background regions) (4) Negative values were substituted with 0. Percentage occupancy on the integrated plasmid (corresponding to the amount of Mediator associated with the burden) was measured as the sum of reads on the entire plasmid sequence from the total amount of reads in each sample.

### Total mRNA

*S. cerevisiae* strains and wild-type *S. paradoxus* were grown overnight at 30^oC^ to OD_600_ ∼0.3. Cell size and count for each sample were individually assayed: Next, the cultures were diluted 1:40 with 0.5M NaCl and immediately measured in Multisizer4 COULTER COUNTER (Beckman Coulter). A fixed amount of ODs of *S. paradoxus* cells was added to twice as many ODs of each *S. cerevisiae* sample, such that the OD ratio between them is constant throughout the samples. The mixed samples were then flash frozen.

RNA extraction and library preparation were performed as described above, and the fastq files were then processed by a pipeline for RNAseq data that was created by Gil Hornung (INCPM, Weizmann Institute of Science, Israel), as described in (Herbst *et al.* 2017). Total reads were normalized to the ratio between the *S. cerevisiae* and *S. paradoxus* sum-of-reads and then to the number of cells as measured in the experiment, as described earlier. Twelve repeats in SC and six repeats in Low Pi/N. Shown is the mean value with +- SE.

### GFP fused library

#### Setup and procedure:

Query strains for screens (Y8205 background; mCherry burdened cells) were constructed on a synthetic genetic array ready strain and were integrated into yeast libraries using the synthetic genetic array method (Tong and Boone 2006; Cohen and Schuldiner 2011). A RoToR bench-top colony array instrument (Singer Instruments) was used to handle libraries (Tong and Boone 2006; Cohen and Schuldiner 2011). Strains from opposing mating types harboring mCherry burden and single GFP fused protein were mated, and diploid cells were selected. Sporulation was induced (by moving the yeast to nitrogen starvation media for seven days), and haploid cells were selected using canavanine and thialysine (Sigma-Aldrich). By moving the haploid cells to plates containing selections for the combination of manipulations desired, a final library containing GFP labeled proteins on the background of low and high burden was created.

For the screening, two 1536 well plates (plates corresponding to the same genes in the control library and the protein burden library) were taken out from the 4°^C^, and a single quarter was replicated separately into 80µl SC 386 well plate. The 386 well plates were left shaking at 30^oC^ overnight. The following day, the plates were diluted 1:80 and mixed 1:3 (low:high burden) using TECAN Freedom EVO^©^ robot. The mixed plate was left shaking at 30^oC^ for ∼4-4.5 hr for a final OD of ∼0.5-1.5. Then the plate was read in FACS in a “high-throughput” mode for GFP and mCherry with an average of ∼50,000 cells.

Two biological repeats of the whole GFP-burden libraries were produced (Figure 5E and S5F).

### Analysis

For each protein in the two biological repeats, cells were divided *In Silico* according to their mCherry level by manual gating, and for each subpopulation, the median GFP was calculated. The ratio of the two libraries was plotted against the control protein abundance, and a trend line was calculated using MATLAB MALOWESS function for the proteins whose fluorescence was above the autofluorescence (∼200[A.U]).

### Data availability

Strains and plasmids are available upon request. The paper’s raw data are available in fileS1 at: https://doi.org/10.6084/m9.figshare.12014937 and fileS2-4 at https://doi.org/10.6084/m9.figshare.12093624.

## Results

### The transcriptional response to protein burden

**Figure 1:**
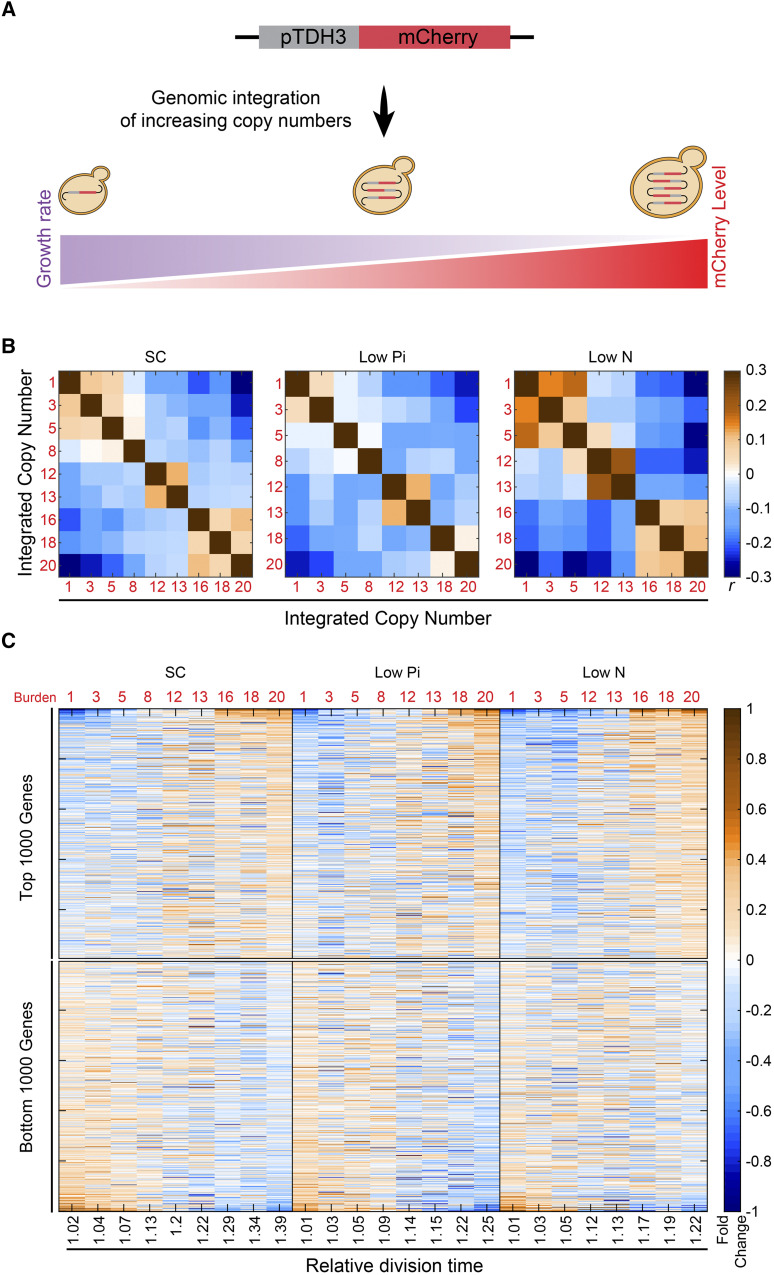
The transcription signature of burdened cells: (A) Engineering libraries of burdened cells: Transforming yeast with a linearized plasmid harboring *TDH3* promoter-driven mCherry results in a variable number of genomic tandem integrations per cell. Individual clones with increasing copy number are taken for further analyses. See methods for details. (B) *Cellular transcription response changes gradually with the level of forced expression:* Shown are the Pearson correlations *r* between the transcription profiles of cells burdened with the indicated mCherry copy number, grown in the indicated conditions. Expression levels were normalized by their mean value in the specific condition and log_2_-transformed. (C) *Expression levels*: The top and bottom 1000 affected genes were selected and sorted by the strongest change with the relative growth rate in standard media SC. The expression is shown as a function of cell growth rate (x-axis) relative to WT, corresponding to the indicated burden copy number.

Cells modify their gene expression when subjected to genetic or environmental perturbations. Often, the expression signature of such cells provides a sensitive probe of the perturbation. Accordingly, the expression signature of cells forced to express excessive amounts of inert mCherry protein could reveal the internal pathways and limitations inflicted by this burden. We previously constructed a library of budding yeast cells, with each strain containing a different copy number (between 1-20 copies) of genomically integrated pTDH3-mCherry constructs. These strains produce mCherry proteins at increasing levels (Figure 1A), peaking at ≈30% of the total cellular proteins for 20 copies. Further, these strains exhibit a linear increase in size concomitant with a linear decrease in growth rate (∼50% and ∼30%, respectively, (Kafri *et al.* 2016)). To define the transcription changes inflicted by the burden, we grew strains with increasing copy number to logarithmic phase and measured their gene expression. We repeated this profiling experiment in three conditions: standard media (SC), media low in nitrogen (Low N), and media low in phosphate (Low Pi). As expected, the overall pattern of gene expression changed gradually with mCherry copy amounts (Figure 1B-C).

### Distinguishing expression changes specific to protein burden from changes common to slow-growing cells

**Figure 2:**
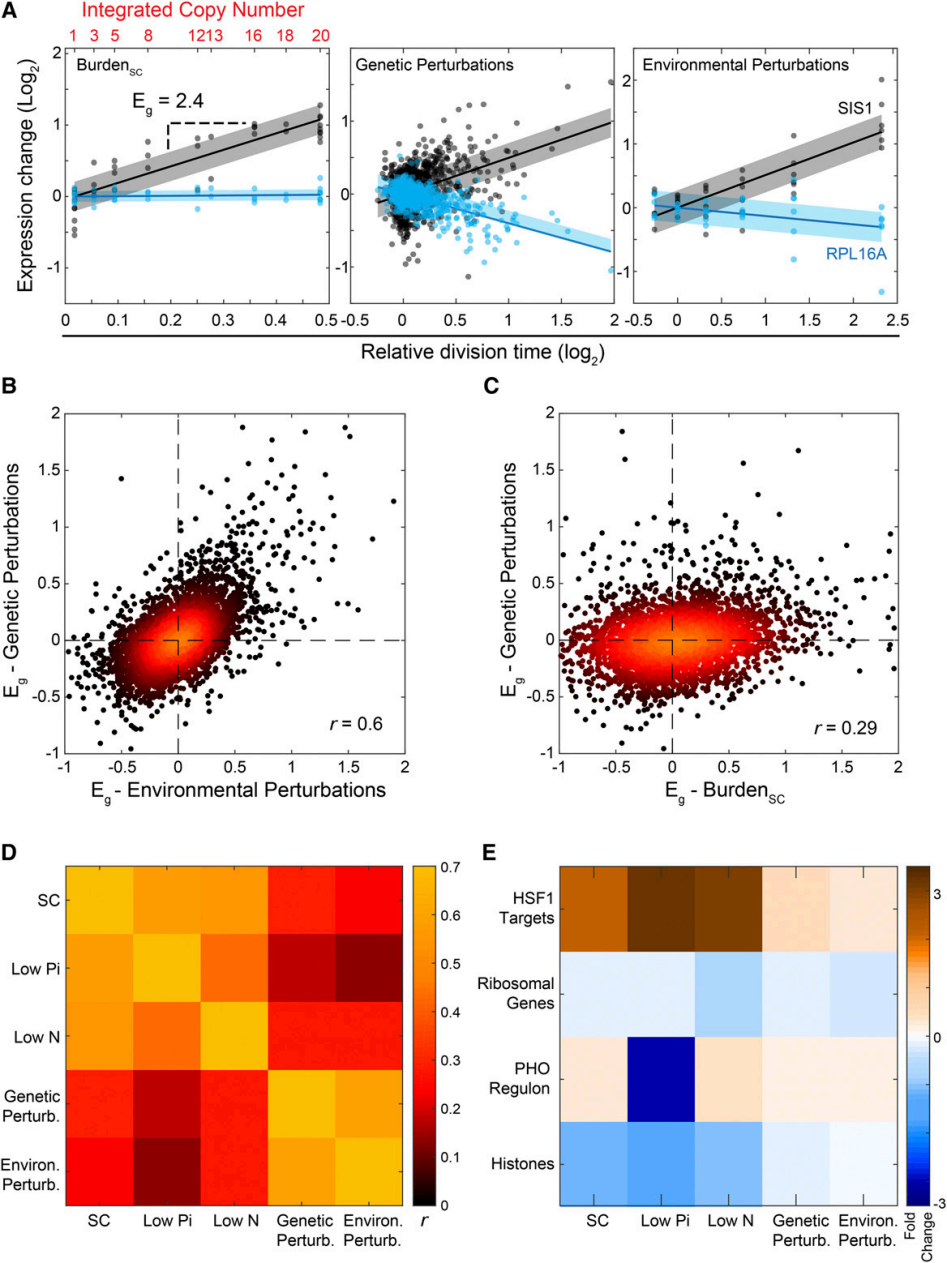
The transcriptional response to protein burden is distinct from the slow growth program: A) Growth-rate response (“E_g_”): Shown are the expression levels of SIS1 (black) and RPL16A (blue) measured in strains of the three indicated datasets as a function of the relative growth rate. The gene-specific growth rate responses are defined by the slope (*E_g_*) of this relation, as indicated. (B-D) *Genome-wide correlations between growth rate responses of burdened cells and perturbed cells:* Shown are the values of the growth rate expression responses for all genes between the specified datasets (B, C), with the Pearson *r* correlation indicated. Pearson correlations between all datasets are shown in (D). (E) *Gene-groups showing a coherent growth-rate response:* The set of genes exhibiting the most significant growth rate response was defined for each dataset. These sets of genes were compared with predefined gene groups associated with a joint function or regulatory properties. Shown are groups with significant enrichment in at least one gene-set (See also Figure S2A).

Previous studies described genes whose expression correlates with the growth rate over a wide range of genetic or environmental perturbations (Hughes *et al.* 2000; Gasch *et al.* 2000; Zurita-Martinez and Cardenas 2005; Regenberg *et al.* 2006; Levy *et al.* 2007; Brauer *et al.* 2008; O’Duibhir *et al.* 2014). Since forced expression of unneeded proteins reduces growth rate in proportion to the added burden (Dong *et al.* 1995; Shachrai *et al.* 2010; Scott *et al.* 2010; Makanae *et al.* 2013; Kafri *et al.* 2016), transcription changes observed in these cells could result from their slow-growth phenotype. To distinguish expression changes that are specific to the burden from those that are general consequences of slow growth, we compared our data to two published compendiums reporting transcription profiles and growth rates. The first dataset described wild-type cells grown in chemostat-based environments (“Environmental Perturbations” (Brauer *et al.* 2008)). The second described 1,484 viable deletion mutants grown in non-stress conditions (“Genetic Perturbations” (O’Duibhir *et al.* 2014; Kemmeren *et al.* 2014)). In each dataset, we defined the degree at which gene expression changes with growth rate (*E_g_*, Figure 2A, Figure S1A). This analysis provided us with three directly comparable gene-specific measures for each dataset (“Burden,” “Genetic,” and “Environmental” perturbations).

The growth-rate responses observed in the genetic and environmental external datasets were highly correlated (Figure 2B). By contrast, the burden response was notably different (Figure 2C-D). Therefore, the majority of expression changes observed in the burdened cells resulted explicitly from the forced production of proteins. To understand these changes, we tested for classes of genes preferentially affected. We checked the enrichment of gene groups defined by GO-slim, binding to the same transcription factors, and co-expression in multiple datasets (Ihmels *et al.* 2002, 2004) (Figure 2E, Figure S2A). Hsf1-dependent chaperones were consistently induced in the burdened cells throughout conditions. However, the induction of this gene group was not unique to the burden but also seen in the other slow-growing perturbations. We did not detect any other group using this enrichment test. In particular, neither GCN4-dependent genes, reporting on amino-acid depletion, nor oxidative-phosphorylation genes, related to energy balance, showed a consistent change with the increasing burden. Indeed, the specific rates of glucose uptake and ethanol production remained invariant to the burden, suggesting that central metabolic fluxes remained mostly unaltered in response to the protein burden (Figure S2B-C).

### Burdened cells correlate with mutants perturbing transcription initiation

**Figure 3:**
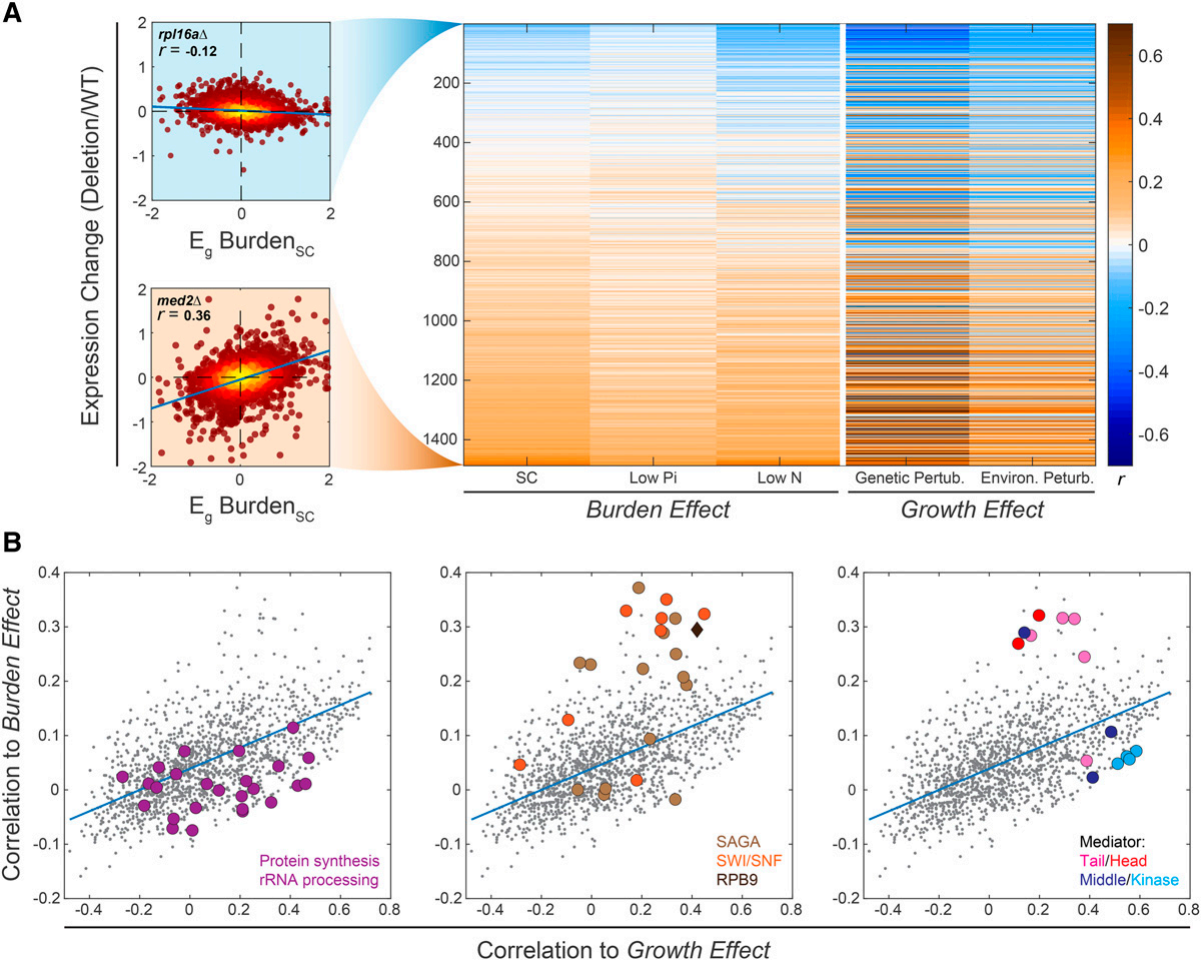
Transcription signature of burdened cells correlates with that of transcription-perturbing mutants: (A) Correlations between the burden response and the transcription response to gene-deletion mutants: Shown are the Pearson r correlations between the growth-rate response E_g_ (measured in the indicated dataset and condition, FileS4), with the transcription signature of each individual gene-deletion mutant. Mutants are ordered by the correlation values with the burden response, averaged over the three conditions. Specific mutants are highlighted, as indicated (see also Figure S3A). (B) Distinguishing mutants that correlate specifically with the burden response: Correlations between mutant signature and burden response (as in A, averaged over the three conditions) are plotted as a function of the correlations between mutant signature and growth-rate response (as in A, averaged over the genetic and environmental responses). Each dot is a mutant, color-coded as indicated. See also Figure S3B.

As a complementary approach to predict cellular processes perturbed in burdened cells, we measured the correlations between the transcription changes caused by the burden and the transcription signatures of the 1,484 gene-deletion mutants (O’Duibhir *et al.* 2014; Kemmeren *et al.* 2014) (Figure 3A “Burden Effect”, Figure S3A)_._ To control for growth-related changes, we also correlated the mutants with the growth-related transcription response, namely the expression changes that correlate with the change in growth rate (Figure 3A “Growth Effect”).

Burdened cells reallocate ribosomes for translating the mCherry protein. This, together with the fine-tuning of ribosome content with growth rate found in wild-type cells, led us to expect that the burdened cells will show a transcription signature that best correlates with that of translation-perturbing mutants, such as deletion of ribosomal components. However, this was not the case: there was little similarity between the transcription signature of the burdened cells and that of translation mutants (Figure 3B, left; Figure S3B-C). The “translational buffer” we have reported (Metzl-Raz *et al.* 2017) might allow cells to partially compensate for the effective (passive) dilution of ribosomes. Thus, cells do not sense significant depletion of the translation machinery, hence the lack of correlation with translation mutants. It may also be that the translation regulatory response is below our detection limit and that the more substantial effect of transcriptional machinery depletion dominates. Correlations between the burden signature and the signature of mutants associated with the protein or mRNA degradation were also low (Figure S3B).

The majority of mutants that correlated most strongly with the burdened cells were associated with gene transcription. These include deletions of RPB9, the only non-essential component of the RNA-Polymerase II profiled in the compendium, and of components of the chromatin-remodeling complexes SAGA and SWI-SNF. Particularly high correlations were found with mutants of the Mediator complex (Figure 3B, middle and right; Figure S3B, D). The Mediator plays a central role in transcription initiation and re-initiation by physically linking specific transcription factors with the general machinery. The mediator complex is composed of a tail sub-complex, which binds Upstream Activating Sequences (UASs) and recognizes particular transcription factors, a head sub-complex, which binds RNA polymerase II, and a middle sub-complex that bridges the head and tail sub-complexes (Sikorski and Buratowski 2009; Ansari and Morse 2013; Allen and Taatjes 2015; Malik and Roeder 2016; Tantale *et al.* 2016; Jeronimo and Robert 2017; Soutourina 2017; Haberle and Stark 2018). An additional inhibitory sub-complex of the Mediator, the Kinase, competes with the RNA polymerase for the same binding site on the Mediator complex and needs to dissociate to allow polymerase binding and transcription initiation (Andrau *et al.* 2006; Aristizabal *et al.* 2013; Gonzalez *et al.* 2014; Clark *et al.* 2015; Jeronimo *et al.* 2016; Petrenko *et al.* 2016). Of the seven subunits of the Mediator tail or head sub-complexes whose signature is available in the compendium, six were correlated with the burden response. By contrast, mutants of the middle or inhibitor kinase sub-complexes showed no such correlation (Figure 3B, right. Except for MED31, whose subunit association is somewhat ambiguous (van de Peppel *et al.* 2005)).

### Phenotypic similarity between burdened cells and mediator mutants

**Figure 4:**
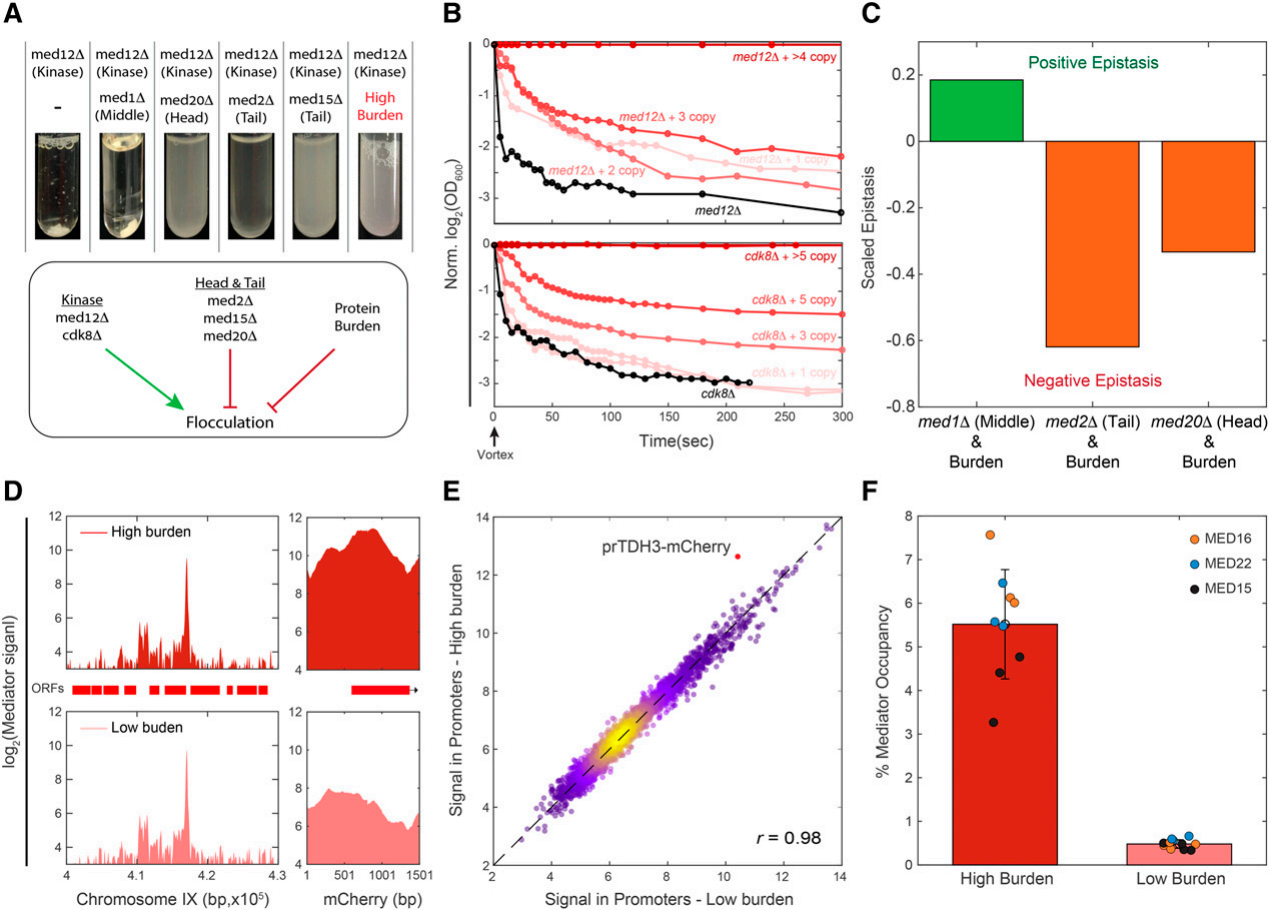
Genetic interaction between burden and Mediator mutants: (A-B) Protein burden phenocopies mutants deleted of mediator head or tail components: Shown are flocculation phenotypes, quantified as described in Methods. Deletion of the mediator kinase subunit MED12 induces flocculation, but this is reverted when deleting components of the head or tail sub-complexes, or by introducing protein burden. Note the gradual effect of increasing burden in this phenotype (B and Figure S4A). (C) Epistatic interactions between burden and mediator mutants: Burden libraries were prepared in the background of the indicated mutants, and growth rates were quantified using sensitive competition assays. Epistatic interactions were defined, according to Segrè *et al.* (2005) (methods). (D-E) ChIP-Seq suggests that binding of the Mediator to endogenous promoters is invariant to protein burden: Genomic binding profiles of the indicated mediator components were profiled in high and low burden strains using ChIP-Seq. Read coverage along chromosome IX (left) and at the mCherry locus (right) are shown in (D), and promoter-averaged binding strengths in the high *vs.* low burden strains are shown in (E). Background signal was removed, see Methods. Note the increased binding to the TDH3-mCherry promoter in the high-burden cells. The fraction of the Mediator that binds to the burden constructs is shown in (F) for the three indicated Mediator subunits in three repeats.

To verify that the similarities in gene expression between burdened cells and mutants of transcription initiation reflect shared internal perturbations, we focused on mutants of the Mediator complex. As a validation of our screen, we re-engineered the respective mutants and profiled their gene expression, verifying the correlation between their transcription signature and that of the burdened cells (Figure S3B-D). Mutants that affect the same process often exhibit epistatic interactions (Elena and Lenski 1997; Lenski *et al.* 1999; Hartman *et al.* 2001; Phillips 2008). To examine whether this is also the case for protein burden and Mediator mutants, we prepared burden libraries in the background of mediator mutants (Figure S4B-C,F). We measured the relative fitness of cells in these libraries and quantified their epistatic interactions using the formalism suggested by Segrè *et al.* (2005). Negative epistasis was observed between the burden and mediator tail or head mutants, consistent with the similarity in their transcription profiles (Figure 4C). Conversely, mutating the middle sub-complex did not result in a negative epistatic interaction and showed a positive (alleviating) interaction with the burden (Figure 4C, Figure S4D).

Next asked whether burdened cells show phenotypes that are similar to those exhibited by mutants of the Mediator’s head or tail sub-complexes. Cells deleted of the Mediator Kinase inhibitory sub-complex are pseudo-hyphal and flocculate when growing in liquid media (Hengartner *et al.* 1995; Holstege *et al.* 1998). This phenotype is reverted by deleting components of the Mediator tail or head sub-complexes, but not by deletion of middle sub-complex components (Palecek *et al.* 2000; van de Peppel *et al.* 2005; Gonzalez *et al.* 2014; Law *et al.* 2015; Jeronimo *et al.* 2016) (Figure 4A). We, therefore, asked whether protein burden will similarly revert the flocculation phenotype of kinase-deleted cells. This was indeed the case: increasing mCherry expression in kinase-deleted cells progressively reduced flocculation (Figure 4A-B, Figure S4A). Therefore, the protein burden phenocopies the mediator tail or head mutant phenotype, consistent with their similarity in gene expression.

Taken together, the pattern of epistatic interactions between burden and mediator mutants is consistent with the similarities in their gene expression profiles and flocculation phenotypes.

### Protein burden does not deplete mediator subunits from endogenous promoters

The similarities in gene expression and phenotypes between burdened cells and mediator mutants may be explained if mCherry production depletes the Mediator from endogenous promoters. To examine this, we measured the genome-wide binding profiles of three Mediator head and tail subunits using ChIP-Seq. Binding patterns at endogenous genes were insensitive to the burden (Pearson Correlation of 0.98 Figure 4D-E, Figure S4E). Further, even in the strains that expressed ≈15 copies of the mCherry gene and showed ≈25% growth defect, only ≈5.5% of detected binding events were localized to the integrated mCherry construct (Figure 4F). The binding levels suggest that the Mediator is not depleted from endogenous promoters, but we cannot rule out this possibility due to the complexities of conventional ChIP (Teytelman *et al.* 2013; Jeronimo and Robert 2014; Paul *et al.* 2015; Hu *et al.* 2015). We also note that our relative ChIP measurements cannot exclude the possibility that the total amount of mediator binding is lower in burdened cells. We find this unlikely, though, as we see no significant decrease in Mediator’s genes expression levels.

### An increase in absolute total mRNA levels in burdened cells leads to a transcription initiation dependent differential expression pattern

**Figure 5:**
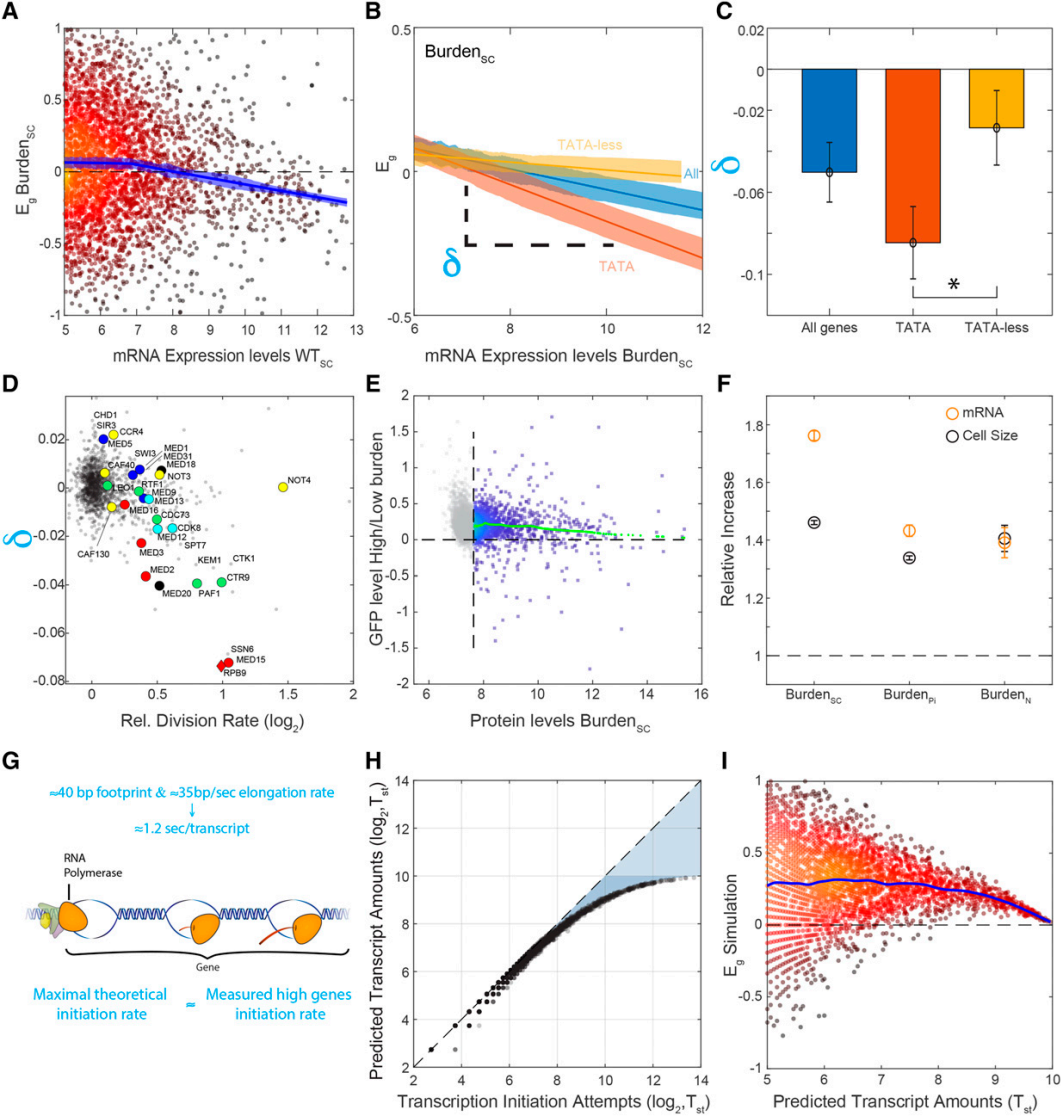
Transcription-promoting feedback activated in burden cells: (A-C) Relative expression of high-abundance genes tends to decrease in burdened cells: Shown are the relative changes in gene expression in burdened cells as a function of absolute mRNA abundance in wild-type cells (A). This effect is accentuated in TATA containing genes (B), with the lines showing the linear fit and the shading the SEM. The expression-dependent bias was quantified by the slopes (δ) of these dependencies. Averaged values of this bias, calculated in the different datasets, are also shown (C). Error bars represent SEM. (D) Mutant strains preferentially affect highly-expressed, TATA-containing genes: The expression-dependent bias (δ) was calculated for each deletion mutant, as in B. Shown are the values of this bias as a function of the mutant growth rate. Genes associated with transcription initiation and elongation are marked. (E) Cells burdened with mCherry production increase their endogenous protein levels: Shown are individual measurements of each protein-GFP fusion in the GFP library in the high burden strain *vs.* low burden. The mean increase in GFP across all informative proteins (above the detection limit marked by the vertical dashed line) is ≈15%. Smoothed data are shown in green using Lowess (malowess, MATLAB 2018a). (F) Burdened cells increase the overall amounts of endogenous transcripts: The total amount of mRNA was measured using sequencing, calibrated by an external spike-in reference. Values from the literature are indicated; see text for details. (G) The maximal possible limit of transcription initiation rate: A new initiation event can only occur once the polymerase has elongated away from its initiation site. This elongation rate, therefore, defines an upper bound on the possible rates of transcription initiation. (H-I) Simulation of the transcription initiation process: The model assumes that initiation attempts are stochastic, characterized by some attempt rate. An attempt is deemed successful if it occurs at a sufficient delay from a previous successful attempt. This delay corresponds to the time required for the polymerase to clear the initiation site. Shown is the frequency of successful initiation events as a function of the attempt rate (H). The consequence of increasing the frequency of the overall attempts, as we assume it happens in burdened cells, is shown in (I), where the blue line is cubic smoothing spline. Note the limited efficiency of this feedback at genes transcribed at high rates.

Mutants of the mediator complex preferentially perturbed the expression of highly expressed and TATA-containing genes (Zenklusen *et al.* 2008; Corrigan *et al.* 2016; Cho *et al.* 2016; Larsson *et al.* 2019). This effect is attributed to the mediator role in transcription initiation and re-initiation, which is expected to be particularly important in highly expressed genes, and in genes that are expressed in rapid bursts, as implicated for TATA-containing genes (Tirosh *et al.* 2006; Contreras-Levicoy *et al.* 2008; Ravarani *et al.* 2016; Urban and Johnston 2018; Wang *et al.* 2019). We reasoned that the same signature characterizes the burden transcriptional response, explaining its similarity with the mediator mutants. This was indeed the case: the relative expression of highly expressed genes, and in particular, those containing TATA in their promoters appeared to decrease in burdened cells as compared to wild-type (Figure 5A-C, Figure S5G). Of note, the same signature was also found in slow-elongating RNA polymerase II mutants (*e.g.*, mutants of the PAF1 transcription elongation complex (Figure 5D, Figure S5C)), but was not a general consequence of slow growth, as this expression signature was not seen in the majority of slow-growing mutants (Figure S5A).

The expression signature we measure defines the relative gene expression, namely the abundance of each transcript relative to that of all other transcripts. The reduction of highly expressed genes in relative expression could indicate their lower relative induction. Alternatively, this signature can be a consequence of a global increase of absolute expression levels, which fails to increase the expression of the highly transcribed genes (Figure S5G). The fact that burdened cells increase in size and in protein content (Figure 5E, Figure S5E-F, (Kafri *et al.* 2016; Metzl-Raz *et al.* 2017)) led us to consider this second possibility. Indeed, previous studies have shown that mRNA abundance scales with cell size in response to different perturbations (Mitchison 2003; Zhurinsky *et al.* 2010; Marguerat and Bähler 2012).

To examine if burdened cells increase the overall abundance of endogenous genes, we compared the total mRNA amounts using an *S. paradoxus* spike-in as a normalization standard. As we hypothesized, total absolute mRNA content in the burdened cells was significantly (≈75%, SC) higher than in wild-type (Figure 5F) and verified the absolute increase we observed for each protein (Figure 5E). Therefore, the majority of gene transcripts increase in abundance in the burdened cells, to the extent that exceeds the size increase of these cells.

### The mechanistic limit restricting transcription rates

Our results suggest a parsimonious explanation for the transcription signature of burden cells: forced protein production increases cell size and concomitantly increases overall transcription capacity. A proportional increase in the abundance of most endogenous transcripts and proteins follows. This increase, however, fails at rapidly transcribed and bursty genes. Our interpretation is that wild-type cells transcribe highly expressed genes at rates that approach the maximal possible limit, and are thus incapable of further increasing their transcription in burdened cells.

We examined the consistency of this model with published data. The rate of transcription initiation is limited by the time required for the polymerase to elongate away from its initiation site (Ehrensberger *et al.* 2013; Choubey *et al.* 2015). Considering the polymerase footprint on DNA (∼35bp) (Brabant and Acheson 1995; Selby *et al.* 1997), this imposes a maximal initiation rate of ∼1.2 sec/transcript, corresponding to an average elongation rate of 2 kb/min ((Edwards *et al.* 1991; Mason and Struhl 2005; Pérez-Ortín *et al.* 2007; Darzacq *et al.* 2007; Swinburne and Silver 2008; Zenklusen *et al.* 2008; Koš and Tollervey 2010; Pelechano *et al.* 2010), FileS2 & FileS3, Figure 5G). Initiation rates are expected to vary widely between genes, depending on their expression levels and burst frequencies, with measured initiation rates available for only a few genes. Still, several of these measurements report initiation rates that are on par with this maximal limit: The *Drosophila* hsp70 transcript, for example, is produced every ∼1.5-3 sec (Lengyel and Graham 1984), similar to the production rate of the Dictostylium Act1 gene during transcription bursts (Corrigan *et al.* 2016). In budding yeast, oxidant-exposed cells produce TRR1 transcript at estimated four-second intervals (Monje-Casas *et al.* 2004). Similar rates were measured for the PDR5 gene during its transcription bursts (Zenklusen *et al.* 2008) and estimated for HIS1 transcripts driven by strong promoters (Iyer and Struhl 1996). Further, estimating initiation rates based on measured values of mRNA abundance and degradation rates are also consistent with these high initiation rates, suggesting that highly expressed genes, and in particular those produced in bursts, are transcribed at rates that approach the theoretical maximum (FileS3).

We next used mathematical simulations to examine if our model of burdened cells can recapitulate the observed signature. Specifically, we simulated stochastic transcription, where an attempt to initiate transcription is successful only if it occurred at a sufficient delay from the previous one, allowing clearance of the polymerase binding site. We then considered genes whose transcription is initiated at different frequencies and measured the frequency of successful events. As expected, the rate of successful initiation events approaches saturation at frequencies significantly lower than the theoretical maximal rate (Figure 5H). Increasing the overall transcription capacity further increases the expression of the majority of genes, but fails at highly expressed gene, recapitulating the transcription signature of burden strains (Figure 5I).

## Discussion

In this work, we set out to determine processes that limit protein synthesis in cells. We approached this by examining the consequences of forcing cells to express high levels of unneeded proteins. Our guiding hypothesis was that processes that are limiting and therefore carried out at maximum capacity in wild type cells would fail to adapt to this increasing demand. To identify such processes, we compared the transcription signature of burdened cells with the respective signatures of hundreds of gene-deletion mutants. We initially expected that the need to translate high levels of mCherry proteins will deplete ribosomes from endogenous transcripts and will, therefore, reflect conditions of insufficient translation, corresponding to deletions of translation factors or ribosome subunits. This, however, was not the case. Rather than translation-perturbing mutants, we found that the burdened cells mostly resemble mutants deleted of components of the general transcription machinery, most notably the head and tail mediator subunits. We examined if this shared signature resulted from the depletion of Mediator subunits from endogenous promoters and found this unlikely as only ∼5% of the bound Mediator localized to the burden constructs and mediator binding to endogenous loci remained invariant. Our data suggest that Mediator is not depleted from endogenous promoters, but we cannot rule out this is a possibility due to the complexities of conventional ChIP (Teytelman *et al.* 2013; Hu *et al.* 2015).

Modeling the transcription process highlighted a limitation of a very different nature: a physical limit that restricts the maximal possible rate of transcription initiation. This limit is set by the polymerase’s molecular properties: its DNA footprint and the rate by which it elongates along the transcript to clear the promoter for another incoming polymerase (promoter clearance). Available data suggest that this limit is relevant for *in-vivo* transcription rates, as highly transcribed genes appear to be transcribed close to this limit (FileS2 & FileS3). We found that protein-burdened cells increase the amount of endogenous mRNA, probably as a consequence of their larger size, caused by perturbed size-regulation. This increase, however, is limited in highly expressed and bursty genes. The transcription signature of burdened cells can, therefore, be explained by their inability to induce further the expression of genes that are already transcribed close to their limit. However, we do not think that the majority of growth defects we describe come from this change in expression. Multiple factors contribute to growth reduction, including the (passive) dilution of ribosome concentration, as discussed in our previous study (Metzl-Raz *et al.* 2017).

Why would cells transcribe genes close to this upper bound of maximal transcription? Could there be a functional benefit in maximizing mRNA production?

**Figure 6:**
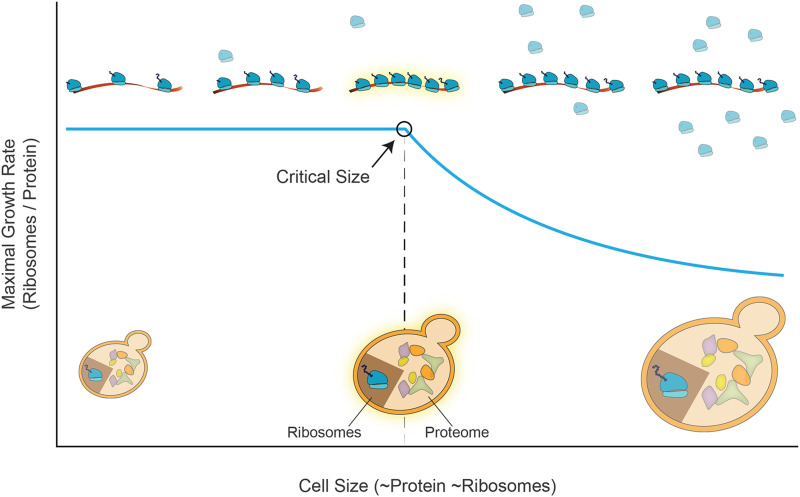
Proposed model for a critical cell size that depends on mRNA transcript abundance during steady-state growth. We suggest a critical cell size above which increasing size (X-axis, directly proportional to protein and ribosome abundance) begins to compete with the optimal growth rate in steady-state conditions (Y-Axis). Given a constant level of mRNA transcripts, by how much can cell size increase while maintaining optimized balanced growth? To maintain optimal growth, the ribosomal fraction scales with the abundance of proteins. As long as it is low enough, the fraction of translating ribosomes can also be maintained, as sufficient mRNA is available. In this regime, the growth rate is not affected by the change in total protein levels. However, further increasing protein abundance beyond this size, necessarily reduces the fraction of co-translating ribosomes, leading to a reduction in cell growth rate. See the text for details.

We suggested that this optimization allows cells to grow in steady-state conditions to maximize their overall protein content (and cell size) while maintaining the internal distribution of proteomic groups that are compatible with optimal growth. Indeed, as part of this optimal growth, the proteome fraction dedicated to translating ribosomes is defined. The ribosomes will be able to translate efficiently only as long as sufficient mRNAs are available as a substrate (Figure 6, below the “Critical Size”). Therefore, maximizing the number of available transcripts defines the number of ribosomes that can simultaneously translate, which in turn, defines the maximal number of cellular proteins (and cell size) compatible with conditions of optimal growth.

Note that the relationship between cell size (protein content) and cell growth relates to evolutionarily optimized conditions. It does not, however, capture changes in cell growth or cell size caused by genetic perturbations. Indeed, the later does not comply with optimal conditions, and accordantly, shows varying relationships depending on the precise perturbation.

Whether cells work close to this limit of maximizing the ribosome number is not clear: Budding yeast expresses an estimated 200,000 ribosomes, compared to 35,000 transcripts (FileS2 & 3 and Miura *et al.*, (2008)). If ribosomes bound all mRNAs at the same efficiency, this would amount to an average of ∼8 ribosomes per mRNA (Arava *et al.* 2003; Zenklusen *et al.* 2008). Considering the footprint of a ribosome on mRNA (∼35bp (Brabant and Acheson 1995; Selby *et al.* 1997)), we expect a rather low ribosome density on most transcripts. However, the extent to which mRNA restricts ribosome numbers should be evaluated based on the highest ribosome densities found at rapidly translated genes. Ribosome densities are higher at gene beginnings, where elongation is slower. Indeed, it was estimated that 20% of ribosomes are positioned adjacent to another ribosome, being detected as a single footprint in ribosome profiling experiments (Diament *et al.* 2018). At least in some transcripts, this high density may argue that ribosome number is adjusted to mRNA abundance, to utilize the available transcripts, and maximize cell size fully.

We note that our model considers conditions of balanced steady-state growth. It has been previously reported that increased cell size (and decreased cell growth rate) are also caused by cell cycle arrest (Zhurinsky *et al.* 2010; Neurohr *et al.* 2019). As these conditions diverge from steady-state growth, we do not expect transcription rates to retain their typical values.

S. Pombe mutants with an increased size grew ∼10% slower, with the transcription rate/protein decreasing by a similar fraction (Zhurinsky *et al.* 2010). Still, the total RNA transcription rate per DNA almost doubled in these large cells. In these conditions, we would indeed expect to see a specific effect on high-expressing genes. This could indicate that s. pombe did not evolve to co-maximize cell size and growth rate (at least in the conditions tested in this paper where wild-type growth is relatively slow). Alternatively, it could be that the microarray technology available at the time was not sensitive enough to observe the relative reduction in the expression of highly expressed genes.

Taken together, we propose that maximizing transcript production, under steady-state and balanced growth, may serve to increase the maximal cell size (or protein content), for which cells can still maintain optimal growth. The maximal possible initiation rate, which limits this production, may, therefore, serve as a fundamental physical constraint, limiting cell size. This is analogous to the time of ribosome translation, which is the fundamental unit defining the cell growth rate. These two physical constraints on transcription and translation, set by the basic biochemical parameters inherent to these processes, may define the characteristic values of the division time and size of rapidly proliferating cells.
